# The Poor-Law Infirmary

**Published:** 1913-03-22

**Authors:** 


### THE POOR-LAW INFIRMARY.

### II.—Fire Risks in Infirmaries—Neglected Precautions.

To superintendents and members of the staff the safeguards against the possible dangers of an outbreak of fire on the institution premises may generally seem sufficient, but it is always well to consider carefully whether these safeguards may not be increased by providing the most up-to-date and effective apparatus. In infirmaries of the old type little exists in the way of really effective preventive means. A few handbuckets, perhaps a fire-extinguisher of antiquated make and very doubtful utility, and half-a-dozen hand grenades, hidden away where they are unlikely to be found in moments of emergency, represent all that the staff has to fall back upon when a fire does break out. In modern hospitals—and the capable infirmary superintendent does his utmost to make his institution approach, as near as possible, to the modern ideal of a well-equipped and regulated hospital—fire risks are never underestimated. Rather are they supposed to be very great risks, and precautions are taken to guard against them by every possible means. While there is still some discussion as to what are the best means to employ in the event of an outbreak, opinion is agreed that only by anticipating what may happen in such an event can the institution come through this most trying of all ordeals without loss of prestige. To neglect such anticipatory training is to court disaster.

On a seagoing vessel one of the captain's most important duties is to see that regular and systematic boat-drill takes place. In an infirmary it is the duty of the superintendent to see that every member of his staff knows exactly what his station is to be when an alarm of fire is given. Perfection can only be attained by practice and drill, and too often such practice and drill are neglected in Poor-Law institutions. The staff has so much to do and there is so little time—these are taken to be sufficient excuses to pardon what is, after all, a grave dereliction of duty. Yet the responsibilities of the staff are very great in this matter. Patients in an infirmary ward are, for the most part, old and infirm; less, perhaps, than patients in a general hospital ward are they able to shift for themselves in an emergency. Nor, indeed, can they be expected to do so. They must rest themselves in the hands of nurses and staff, and it is imperative that these, their guardians, should realise to the full the great responsibility that rests on the institution.

While insisting on fire-drill, practice with the hose and escape, and constant training of the nursing staff in the best methods of emptying the wards when suddenly called upon to do so, the superintendent should make a point of seeing that he has the best support in his endeavours to keep his institution at a high pitch of efficiency. If his is an old building, he should consult with the architect and devise ways in which additional precautions can be taken by rendering certain portions of the building fireproof. If it is a new structure, he will probably find that these essentials have not been overlooked. Nowadays all institutions of this kind are built with fireproof floors, ceilings, and walls, but it is quite possible that considerations of economy may have caused Guardians or architects to adopt inferior methods, and thereby to have added to the risks. The superintendent ought to know the value of his building; he ought to know what part of it is fireproof and what part is not, just as he ought to know what directions the drains take, what is the quality of the flooring, and how the building is heated and ventilated.

### A Neglected Precaution.

A very necessary, but often neglected, precaution is the fire staircase. The mistake is often made of building this too steep and too narrow; a serviceable fire staircase should be wide enough to permit of two nurses carrying comfortably down it a patient lying on one of the ordinary ward beds. If this test is carried out it will be found that several of our institutional staircases fail, in so far as they are so steep that the angle of the bed is inconveniently high, or in that they are so narrow that only the most intricate manœuvring can make the bed pass. A further point to look at is the emergency exit. This should open flush with the ward floor; to have a step here is to court disaster when the exit has to be hurriedly used. It is a counsel of perfection to say that the exits must all open outwards, for, simple as this arrangement seems, it is one that is very often totally neglected. The staircase should have a proper and fairly high guard rail, and should be divided into several stages with large and adequate landings on which in case of need more than one bed may be deposited. Needless to say, it must be strong enough to support the weight of beds and attendants. When a sudden rush occurs, and patients are bundled out, it is quite possible for the landings to become choked while other patients are still being brought downstairs; in such an event the strain on the staircase is great, and it must be made to stand such strain.

Hydrants and water-supply mains should be carefully charted, and replicas of the charts should be fixed in each ward and in the corridors, so that by constantly having these details before their eyes the members of the staff may become thoroughly familiar with the position of every hydrant. It may be argued that this is primarily the duty of the outdoor staff, but it is as well to be on the safe side and to train the nursing staff as well. Nor is it superfluous to make the nursing staff acquainted with the simpler forms of checking an outbreak of fire. How to screw on the hose and how to direct its stream of water to the best advantage are matters of detail which can be easily learned with a little practice; the knowledge and the experience derived from such training give assurance.

Considerable thought must be given to the fire-drills themselves. It is perfectly useless to order such a drill a week beforehand. While organised drills and practices with the fire escapes and with the methods of emptying the ward in the best and safest way are excellent, the real test of efficiency is to be found in the unexpected call which comes when no one is on the look out for it. The difficulty is to make such unexpected alarms without unduly disturbing the patients; with a little care this may easily be avoided, and unexpected drills need not be called when there are in the wards patients who are likely to be seriously affected by an alarm. But the essence of fire-drill is that it is emergency practice for an emergency. The ringing of a fire alarm, or some other signal, should be the first indication given to everyone concerned that it is intended to hold such a drill. Once that signal has been given, everyone should be at his or her station. fully prepared to do what he or she has been told off to do. Defaulters should be punished, and be encouraged to realise their responsibilities as working members of the community of the institution. At such a drill everything should be carried out with quickness and calmness; there should be no hurry without a definite object in view, but the procedure should be thorough to the verge of meticulousness. Very often in institutions fire-drill is a perfunctory matter which is over in a few minutes. Such slipshod methods can only encourage difficulties when the real test comes.

We have already stated that everyone should have his or her special duty. Here again we may take an example from boat-drill at sea. The various members of each boat's crew know exactly what their duties are. A list of names of each crew is kept prominently posted up, and at the weekly practices every member of a boat's crew turns up ready to take his share of the work without, through ignorance or officiousness, interfering with that of his fellow.

That should be the case in institutions. A list of workers in each ward must be posted up, and every person on that list must know definitely and thoroughly what he or she is expected to accomplish when the time comes. The member of the staff in charge of the ward will be told off to act as general supervisor of that ward; the sister-in-charge will superintend the removal of the patients, and each nurse or attendant will have his or her special post, which at all costs must be kept. Where special patients need special care a nurse must be told off to look after them, and she must give her whole attention to such patients until they are brought into a place of safety. The care of the patients is the first consideration, and fire-drill should in the first place be directed to secure the safety of the inmates of the wards, though it is also highly desirable to have special drills in various special parts of the buildings. For instance, one drill every quarter or month may be given to the nurses' quarter, another to the residents' quarter, and so on. But in whichever quarter it is carried out it must be carried out with thoroughness.

